# Social determinants of health and upper gastrointestinal cancer outcomes in the United States: a systematic review

**DOI:** 10.3389/fpubh.2024.1477028

**Published:** 2024-11-19

**Authors:** Brenda Santellano, Rashi Agrawal, Gabriela Duchesne, Muhannad Sharara, Gagan Agrawal, E. Andrew Balas, Meng-Han Tsai, Asha Nayak, Jorge E. Cortes

**Affiliations:** ^1^Georgia Cancer Center at Augusta University, Augusta, GA, United States; ^2^Medical College of Georgia, Augusta University, Augusta, GA, United States; ^3^School of Computing, University of Georgia, Athens, GA, United States; ^4^School of Public Health, Augusta University, Augusta, GA, United States; ^5^Georgia Prevention Institute, Augusta University, Augusta, GA, United States

**Keywords:** cancer, gastrointestinal, gastric cancer, survival, social determinants, healthcare system

## Abstract

**Introduction:**

Social determinants of health (SDOH) are the conditions in which individuals are born, grow, work, live, and age.

**Methods:**

We examined the literature on the association between SDOH and survival of patients with gastrointestinal (GI) cancer [esophageal, duodenal and gastric cancer (GC)] in the United States from 2001 to 2022.

**Results:**

From 38,654 studies across COCHRANE, EMBASE, SCOPUS, WEB OF SCIENCE, and PubMed, we identified 14 relevant studies focusing on GI cancer using the PRISMA flowchart. Eight of the 12 GC studies specifically focused on gastric adenocarcinoma (GAC), the most common histologic subtype. Uninsured patients had a significantly worse overall survival probability. For patients with GI cancer, the highest income level (i.e., in the highest quartile) was associated with improved survival. Being unmarried had a negative impact on overall survival. Overall, people with insurance, higher incomes, and who were married had better overall survival rates.

**Discussion:**

Our findings suggest a clear association between SDOH and survival for patients with GI cancers. However, there is great variability in the factors studied and how these are measured and reported. A better understanding of SDOH is needed to design strategies with an aim to improve patient outcomes.

**Systematic review registration:**

https://www.crd.york.ac.uk/prospero/, PROSPERO (CRD42022346854).

## 1 Introduction

It is projected that in 2040 there will be approximately 28.4 million new cases of cancer worldwide, representing a 47% increase from 2020 (1). Importantly, in high-income countries, the more vulnerable populations are often the most exposed (1). Predictions are that in 2024 there will be 353,820 new cases of gastrointestinal (GI) cancers diagnosed, and 174,320 individuals will die of GI cancers in the United States (US). Of these, esophageal cancer is anticipated to account for 22,370 new cases, gastric cancer for 26,890 cases, and small intestine cancers, including duodenal cancers, for 12,440 cases, all contributing significantly to the overall burden (2). Globally, the incidence of gastric cancer (GC) has decreased from 1990 to 2017, with this reduction largely attributed to economic development and rising Socio-demographic Index (SDI) levels. The lowest incidence rates were observed in southern sub-Saharan Africa (5.2 [5.0–5.4]), eastern sub-Saharan Africa (6.4 [5.9–6.8]), and high-income North America (6.5 [6.3–6.7]) (3).

The incidence of GC, particularly gastric adenocarcinoma (GAC), is higher in non-White racial and ethnic groups compared to the non-Hispanic White (NHW) population in the US (4). The highest incidence rates are observed among major racial and ethnic groups, notably non-Hispanic Black Americans, Hispanic Americans, and Asian Americans. Hispanic men and non-Hispanic Black (NHB) men have a 3.6-fold and 2.9-fold higher incidence of GAC, respectively, compared to NHW men. Among Asian American ethnic groups, the incidence of GAC is up to 14.5 times greater compared to NHW men, with similar trends observed women in these racial and ethnic populations (4, 5). Between 2000 and 2019, GC mortality declined significantly across populations at the national level in the United States. With the fastest declines being observed among both Asian populations (48.3%) and black populations (42.6%). Despite these declines, mortality rates remained statistically significant among minority populations at the county level (6). Disparities in GC mortality among racial and ethnic groups persist, with reports indicating that minority groups experience a two-fold higher mortality risk compared to other populations (7).

In 1998, the WHO launched a campaign for public health action on Social Determinants of Health (SDOH) (8), leading to the creation of the Commission on SDOH, which issued the first recommendations on measuring and addressing SDOH (9). SDOH are defined as conditions in which people are born, grow, work, live, and age, and include the wider set of forces and systems shaping the conditions of daily life (10). The Healthy People 2030 initiative divides SDOH into 5 domains: Economic Stability, Education Access and Quality, Health Care Access and Quality, Neighborhood and Built Environment, and Social and Community Context (11, 12).

There is growing evidence of the impact of SDOH on cancer outcomes (13), with a higher cancer mortality rate in the most underprivileged groups (14). Although prior studies have examined the association between SDOH and GI cancer survival, none of these used a comprehensive framework (as recommended by Healthy People 2030) (11). Because several SDOH components are highly correlated, studies using a comprehensive framework are needed. To address this gap, we performed a systematic review of the literature, examining the association between SDOH and GI cancer survival. The goal of this study is to consolidate the diverse existing literature on SDOH in GI cancer into a homogeneous classification framework. This approach aims to enhance understanding of the impact of SDOH on patients with GI cancers in order to guide future studies to better address healthcare disparities among these patients.

## 2 Materials and methods

This systematic review is registered in PROSPERO (CRD42022346854).^1^ It strictly follows the Preferred Reporting Items for Systematic Reviews and Meta-Analyses 2020 (PRISMA) statement (15) and aligns with the Cochrane Handbook for Systematic Reviews of Interventions (16).

A comprehensive search was performed in the COCHRANE, EMBASE, SCOPUS, WEB OF SCIENCE, and PubMed databases from 2002 to 2022. Additional manuscripts were searched in Google Scholar and in the references included. This 20-year time frame was selected to provide a balance of historical perspective and relatively recent data since social determinants have evolved over time. By limiting our search to this specific period, we aim to provide a concise and focused synthesis of literature that is both manageable and highly relevant to contemporary research questions (Supplementary Table 1).

**Figure 1:**
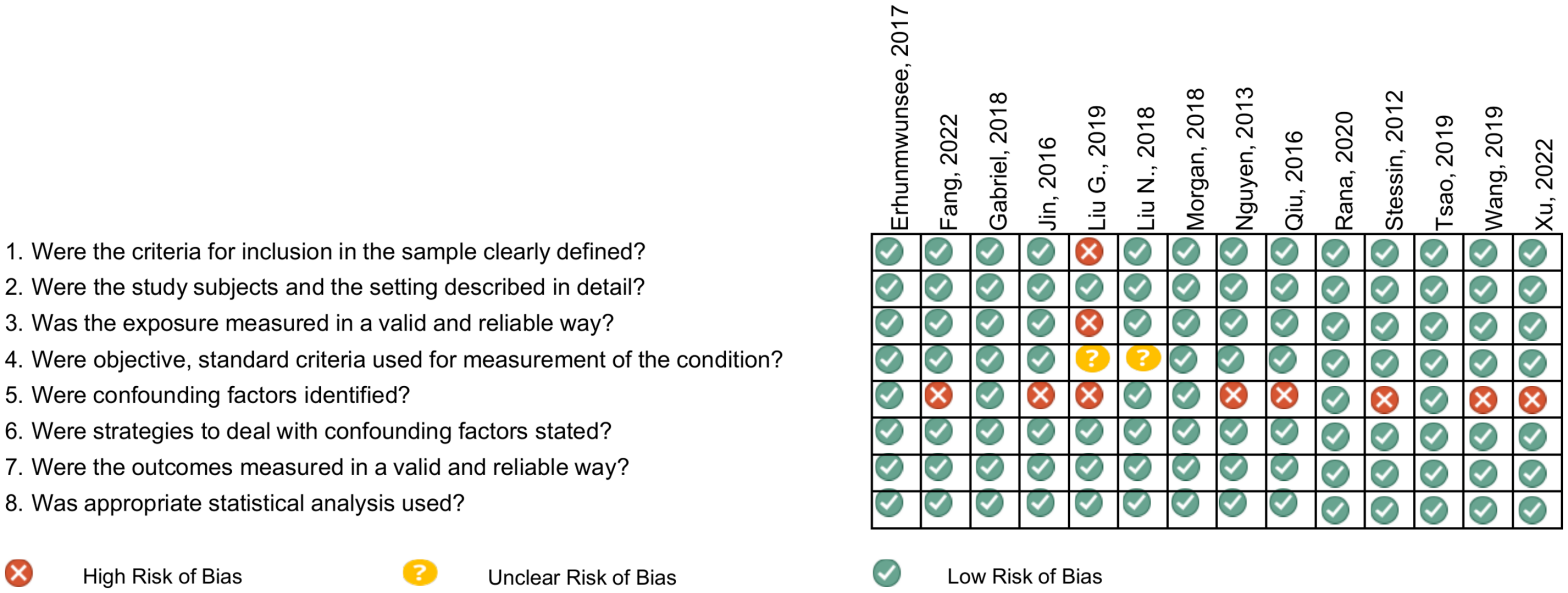
Risk of bias assessed by the Joanna Briggs institute critical appraisal tools for analytical cross-sectional studies.

Selected manuscripts reported US population-based observational studies on the effect of SDOH on GI cancer survival outcomes, aligned with the five Healthy People 2030 SDOH core domains (11). Studies examining colorectal cancer (CRC) were excluded due to its high incidence, since it might dominate the results. Instead, we focused on the less common GI cancers and will analyze CRC separately in a future analysis. Non-cancer studies without SDOH evaluation or outcomes related to cancer survival and general cancer studies without identifiable outcomes by cancer type were excluded. Duplicated publications, those published outside our specified cut-off date, non-English publications, revisions, letters to the editor, pre-clinical reports, and case series reports were also excluded. Eligible manuscripts identified based on the listed search criteria (Supplementary Table 1) were retrieved and imported to Rayyan software. Manuscripts were screened and duplicates removed. Two authors (BS and MS) assessed the relevance of the remaining manuscripts based on abstract and title, with disagreements resolved by consulting oncology experts (JC and AN). Manuscripts not meeting the eligibility criteria were excluded. Manuscripts addressing SDOH and cancer survival were initially selected (*n* = 758), and within this group, 20 studies specific to GI cancers were identified. In the second review stage, the full manuscripts were examined, and exclusion criteria applied (Supplementary Table 2). All references included after the abstract screening were independently assessed by two reviewers (RA and GD) for methodological quality and were evaluated for risk of bias assessment using the Joanna Briggs Institute (JBI) checklist, which comprises eight items and an overall assessment (Figure 1).

**Figure 2:**
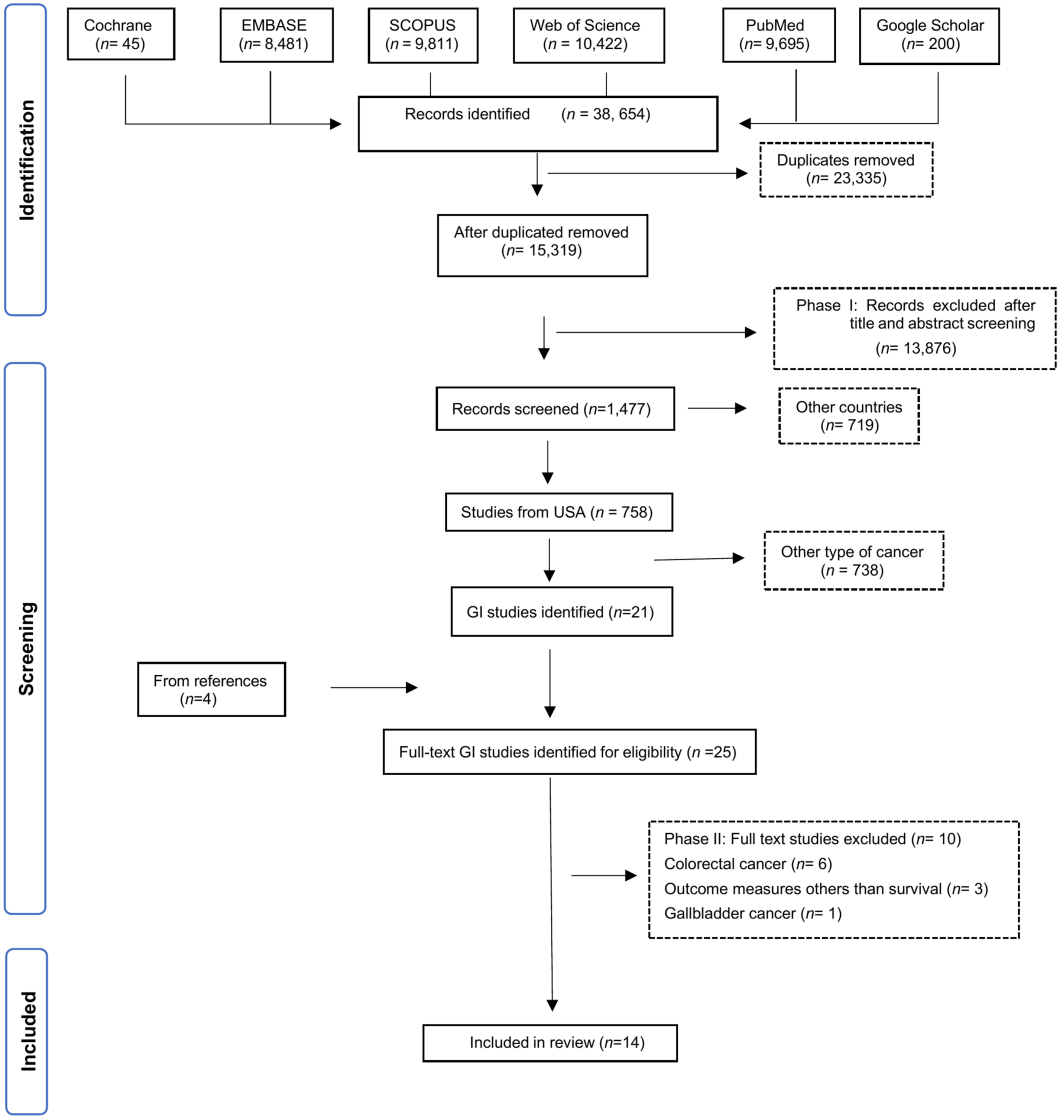
Flowchart of the studies selection process.

A total of 14 eligible manuscripts were identified for this review (Figure 2). Eligible studies were uploaded to Nvivo software to enhance the rigor and depth of the qualitative analysis. Extracted data included first author, publication year, study location, database details, study type, patient characteristics (sample size, population sample, age group), aims, SDOH studied, associated factors, limitations and barriers, and results. Survival measures included overall survival (OS), cancer-specific survival (CSS) and progression-free survival (PFS).

We qualitatively synthesized data on the association between SDOH and GI cancer survival, considering each SDOH as an isolated variable. Study characteristics and SDOH data were extracted (Tables 1, 2), and significant results, including *p* values, were extracted (Tables 3–6). Statistically significant adjusted values, accounting for confounding factors such as age, sex, and ethnicity, were considered.

**Table 1:** Population characteristics of included studies assessing SDOH for gastrointestinal (GI) cancer patients (*n* = 14).

Author (year), Region	Data source. Years	Population sample	Patients N	Age (y.o.)	Cancer type
Nguyen (2013), USA	SEER. 1980–2009	Patients were included if they had a single, primary diagnosis of gastric adenocarcinoma.	42,187	<40^‡^ 40–64 65	Gastric^#^
Xu (2022), USA	SEER. 2007–2016	Diagnosed with GAC, first primary tumor, <25 years at diagnosis cases were e excluded.	30,409	≤65 >65	Gastric^#^
Liu N. (2018), USA	SEER. 2001–2009	Patients diagnosed with regional gastric cancer, limited to patients with histologically confirmed cancer who were continuously enrolled in Medicare parts A and B in the year prior to diagnosis.	5,972	≥65	Gastric
Liu G. (2019), USA	SEER. 1973–2014	All the malignant gastric cancers in our study have been confirmed through pathologic examination.	11,6,718	<50 50–64	Gastric
				65–79	
				≥80	
Morgan (2018), Boston, MA	BMC. 2004–2017	Patients diagnosed with or treated for gastric cancer at an urban, tertiary academic medical center. Patients with GAC not involving the gastroesophageal junction.	249	<65 >65	Gastric^#^
Stessin (2011), USA	SEER. 1998–2007	Patients with surgically resected GAC.	7,348	65.9^‡×^	Gastric^#^
Tsao (2019), Memphis, TN	Multi-hospital healthcare system affiliated with the UTHSC. 2003 − 2018	Initial pathologic diagnosis of GAC made within our institution and age > 18 years. Exclusion criteria were patients with Siewert type I and II gastroesophageal junction tumors, any patient with a first-degree relative with gastric cancer, patients from known kindred with hereditary diffuse gastric cancer syndrome, and ethnicity other than African American or Caucasian.	359	>18	Gastric^#^
Qiu (2016), USA	SEER. 2004–2012	GAC patients were enrolled for study.	29,074	67^×^	Gastric^#^
Jin (2016), USA	SEER. 2004–2010	Age was limited to 18 years or older, and patients with unknown marital status were excluded. The histologic types consisted of adenocarcinoma Mucinous adenocarcinoma, and signet ring cell carcinoma.	18,196	≥18	Gastric
Rana (2020), USA	NCDB. 2004–2013	Patients diagnosed with GAC, from all stages.	88,246	_70_‡×	Gastric^#^
Fang (2022), USA	SEER. 2007–20,016	Diagnosis of LAGC (TNM stage Ib-IIIc), aged 18–64 years at the time of diagnosis, reception of surgery, and complete insurance record.	5,860	18–64^‡^	Gastric
Gabriel (2018), USA	NCDB. 2004–2013	Patients were selected for both Esophageal and Gastric Cancer, with adenocarcinoma histologies for gastric cancer and squamous cell histology for esophageal cancer.	17,547	62.81^×^	Gastric^#^ and Esophageal
Erhunmwunsee (2017), USA	NCDB. 2003–2011	Patients with no metastatic, squamous cell, or adenocarcinoma esophageal cancer diagnosed who underwent an esophagectomy.	11,599	>18 <85	Esophageal
Wang (2019), USA	SEER. 2004–2015	Patients diagnosed with adenocarcinoma of the duodenum, Primary cancer site, excluded if the age at diagnosis was <18 years.	2,018	≤58^‡^ 59–75 >75	Duodenal

BMC, Boston Medical Center; GAC, Gastric Adenocarcinoma; GC, Gastric Cancer; LAGC, Locally Advanced Gastric Cancer; MA, Massachusetts; NCDB, National Cancer Data Base; SDOH, Social Determinants of Health; SEER, Surveillance, Epidemiology, and End Results; TN, Tennessee; USA, United States of America; UTHSC, University of Tennessee Health Science Center; y.o., years old.

# Studies analyzing specifically gastric adenocarcinoma.

‡ Groups according to the age at diagnosis.

× Mean age.

**Table 2:** SDOH according to the Healthy People 2030 framework assessed in gastrointestinal (GI) cancer patients of included manuscripts (*n* = 14).

Author (year), Region	Domain	Category	Group	Objective
Nguyen (2013), USA	HAQ	Treatment access	Treatment	Our objectives were to explore how gastric cancer rates, receipt of care, and outcomes are affected by age, poverty, and acculturation.
			No treatment	
	ES	Poverty rate	Low	
			Medium	
			High	
Xu (2022), USA	HAQ	Insurance status	Insured	We aimed to demonstrate the associations between education level and clinical outcomes inpatients with GAC.
			Uninsured	
			Medicaid	
	ES	Income	Q1-Q4 (Lowest-Highest)	
		Poverty rate	Q1-Q4 (Lowest-Highest)	
	EAQ	Education level	Q1-Q3 (Lowest-Highest)	
Liu N. (2018), USA	HAQ	Treatment access	Treatment	We sought to identify factors associated with undertreatment of regional gastric cancer in this population, as well as to assess overall survival in the undertreated population.
			No treatment	
Liu G. (2019), USA	HAQ	Treatment access	Surgical compliance	We aimed to identify the risk factors associated with surgical compliance and investigate the difference in survival.
			Non-surgical	
			Surgical non-compliance	
Morgan (2018), Boston, MA	HAQ	Insurance status	Medicare	In this paper, we analyze the experience of patients treated for GC at an urban safety-net hospital that serves a diverse, largely foreign-born population.
			Medicaid	
			Non-Federal	
			Other	
	ES	Poverty rate	0–12.9%^#^	
			13–20%	
			20.1–25%	
			>25%	
			Unknown	
Stessin (2011), USA	ES	Poverty rate	% Below poverty line	The aims of this study were to identify demographic factors associated with omission of adjuvant RT and assess the impact of this omission on survival.
		Income	% Median household income	
	EAQ	Education level	% No high school education	
Tsao (2019), Memphis, TN	HAQ	Insurance status	State-sponsored/none	This evaluation was intended to identify differences in disease presentation and delivery of therapy to better understand how potential disparities might be addressed at the local level.
			Private	
			Medicare	
	ES	Income	≤50th percentile	
			>50th percentile	
Qiu (2016), USA	HAQ	Insurance status	Insured	In the present study we analyze the survival difference among different marital status in the United States in GAC patients.
			Uninsured	
	SCC	Marital status	Married	
			Unmarried	
Jin (2016), USA	SCC	Marital status	Married	The relation between marital status and incidence of metastasis at diagnosis, receipt of surgery, and CSS in the group of 18,196 GC patients.
			Single (never married)	
			Divorced/separated	
			Widowed	
Rana (2020), USA	HAQ	Insurance status	Uninsured	The purpose of this study was to investigate the geographic and sociodemographic disparities in GAC patient.
			Insured	
Fang (2022), USA	HAQ	Insurance status	Private Insured	We aim to identify definite association of health insurance status with CSS and OS in LAGC population
			Uninsured	
			Any Medicaid	
			Insured/No specifics	
Gabriel (2018), USA	HAQ	Hospital volume	Low volume	The purposes of this study were to identify racial and/ or socioeconomic disparities among centers performing major surgery for esophageal or gastric cancer, stratified by case volume, and determine the association of these disparities with long-term OS.
			Middle volume	
			High volume	
		Insurance status	Not insured	
			Private	
			Medicare	
			Medicaid	
			Other	
		Facility type	Community cancer program	
			Comprehensive Academic	
			Integrated	
	ES	Income	< $30,000	
			$30,000–34,999	
			$35,000–45,999	
			> $46,000	
	EAQ	Education level	<14%	
			14–19.9%	
			20–28.9%	
			>29%	
Erhunmwunsee (2017), USA	HAQ	Insurance status	Private	This study examines this racial disparity in conjunction with socioeconomic status (SES) and explores whether race-based outcome differences exist.
			Not private	
		Facility type	Community or other	
			Academic	
	ES	Income	Q1-Q4 (Lowest-Highest)	
	EAQ	Education level	Q1-Q4 (Lowest-Highest)	
Wang (2019), USA	SCC	Marital status	Married	The aim of the present study was to elucidate the effects of marital status on the prognosis of patients with duodenal adenocarcinoma.
			Unmarried	

EAQ, Education Access and Quality; ES, Economic Stability; SCC, Social and Community Context; GAC, Gastric Adenocarcinoma; GC, Gastric Cancer; HAQ, Healthcare Access and Quality; USA, United States of America; MA, Massachusetts; SDOH, Social Determinants of Health; TN, Tennessee; Q, Quartile.

# Percentage of population living in poverty measured by poverty level of zip code.

**Table 3:** Results of included studies assessing healthcare access and quality by tumor type (*n* = 11).

Author (year)	Cancer type	Category	Group	HR (95%-CI)	*p-*value
Nguyen (2013)	Gastric^¥^	Treatment access	Cancer-direct surgery	Ref.	
			No cancer-direct surgery	0.75 (0.64–0.86)	0.0001
Xu(2022)	Gastric^¥^	Insurance status	Insured	Ref.	
			Uninsured	1.063 (0.994–1.136)	0.075
			Medicaid	1.062 (1.024–1.101)	0.001
Qiu (2016)	Gastric^¥^	Insurance status	Insured	Ref.	
			Uninsured	1.08 (0.88–1.31)	0.472
Liu N. (2018)	Gastric	Treatment access	Treatment	Ref.	
			No treatment	1.43 (1.32–1.55)	< 0.001
Liu G.(2019)	Gastric	Treatment access	Surgical compliance	Ref.	
			Surgical non-compliance	3.49 (3.43–3.56)	<0.0001
			Non-surgical		
			Surgical non-compliance	Ref.	
				1.08 (1.06–1.10)	<0.0001
Morgan (2018)	Gastric^¥^	Insurance status	Medicare	Ref.	
			Medicaid	-	
			Non-federal	2.00 (1.00–4.02)	0.051
			Other	-	
Rana (2020)	Gastric^¥^	Insurance status	Insured	Ref.	
			Uninsured	1.32 (1.26–1.38)	<0.01
Fang (2022)	Gastric	Insurance status	Private insured	Ref.	
			Uninsured	1.26 (1.03–1.53)	0.02
			Any Medicaid	1.14 (1.02–1.27)	0.02
			Insured/No specifics	1.13 (0.99–1.29)	0.06
Tsao (2019)	Gastric^¥^	Insurance status	State-sponsored/non	1.07 (0.80–1.41)	0.663
			Private	-	
			Medicare	1.21 (0.73–2.02)	0.457
Gabriel (2018)	Gastric^¥^	Insurance status	Not insured	Ref.	
		Low volume^•^	Private	0.88 (0.75–1.04)	0.013
			Medicare	0.99 (0.84–1.17)	
			Medicaid	0.98 (0.81–1.19)	
			Other	0.85 (0.62–1.16)	
	Gastric^¥^	Facility type	CCCP	Ref.	
		Low volume^•^	Integrated	0.90 (0.80–1.01)	0.011
			Academic	0.87 (0.79–0.96)	
			Comprehensive	0.94 (0.86–1.03)	
	Esophageal	Insurance status	Not insured	Ref.	
		Low volume^•^	Private	0.84 (0.66–1.08)	0.009
			Medicare	0.98 (0.76–1.26)	
			Medicaid	1.01 (0.76–1.35)	
			Other	0.99 (0.67–1.46)
	
		Middle volume^•^	Not insured	Ref.	
			Private	0.46 (0.26–0.81)	0.002
			Medicare	0.55 (0.30–0.99)	
			Medicaid	0.84 (0.44–1.63)	
			Other	0.63 (0.28–1.43)	
		High volume^•^	Not insured	Ref.	
			Private	0.37 (0.21–0.67)	0.007
			Medicare	0.33 (0.18–0.60)	
			Medicaid	0.48 (0.23–1.01)	
Other	0.36 (0.15–0.87)		
Erhunmwunsee (2017)	Esophageal	Insurance status	Not private	Ref.	
			Private	0.849 (0.801–0.900)	0.002
		Facility type	Academic Facility	Ref.	
			Community or other	1.094 (1.040–1.151)	0.007

CCCP, Comprehensive Community Cancer Program; HR, Hazard Ratio; Ref., Reference. •Hospital by case volume-low: 1–99 cases per decade, middle: 100–200 cases per decade, and high: >200 cases per decade.

¥ Studies analyzing specifically gastric adenocarcinoma.

**Table 4:** Results of included studies assessing economic stability by tumor type (*n* = 7).

Author (year)	Cancer type	Category	Group	HR (95%-CI) or Months	*p*-value
Nguyen (2013)	Gastric^¥^	Poverty rate	Low	–	
			Medium	–	
			High	1.10 (1.00–1.21)	0.04
Xu (2022)	Gastric^¥^	Income	Q1^a^	Ref.	
			Q2	0.944 (0.896–0.993)	0.027
			Q3	0.938 (0.880–1.000)	0.05
			Q4^b^	0.902 (0.829–0.981)	0.016
Morgan (2018)	Gastric^¥^	Poverty rate	<20%^c^	24^d^	0.006
			>20%	16	
Stessin (2011)	Gastric^¥^	Poverty rate	% Below poverty line	1.007 (0.983–1.031)	0.0306
		Income	Median household	0.996 (0.989–1.002)	0.0306
			income		
Tsao (2019)	Gastric^¥^	Income	<50th percentile	1.05 (0.60–1.83)	0.859
			>50th percentile	-	
Gabriel (2018)	Gastric^¥^	Income	< $30,000	Ref.	
		Low volume^•^	> $46,000	0.87 (0.80–0.94)	<0.001
			$35,000–45,999	0.93 (0.86–1.01)	
			$30,000–34,999	0.99 (0.90–1.09)	
		Middle volume^•^	< $30,000	Ref.	
			> $46,000	0.87 (0.65–1.15)	<0.001
			$35,000–45,999	1.32 (1.00–1.76)	
			$30,000–34,999	1.33 (0.98–1.81)	
Erhunmwunsee (2017)	Esophageal	Income	Q1^a^	Ref.	
			Q2	0.960 (0.897–1.046)	1
			Q3	0.870 (0.806–0.940)	0.004
			Q4^b^	0.803 (0.743–0.867)	< 0.0001

CI, Confidence Interval; HR, Hazard Ratio; Q, Quartile; Ref., Reference. •Hospital by case volume-low: 1–99 cases per decade, middle: 100–200 cases per decade, and high: >200 cases per decade.

¥ Studies analyzing specifically gastric adenocarcinoma.

a Lowest.

b Highest.

c Percentage of population living in poverty.

d Months.

**Table 5:** Results of the included studies assessing education access and quality by cancer type (*n* = 4).

Author (year)	Cancer type	Category	Group	HR (95%-CI)	*p*-value
Xu (2022)	Gastric^¥^	Education level	Q1^a^	Ref.	
			Q2	0.926 (0.885–0.969)	0.001
			Q3^b^	0.915 (0.860–0.973)	0.005
Stessin (2011)	Gastric^¥^	Education level	% No high school education	0.998 (0.987–1.010)	0.0306
Gabriel (2018)	Esophageal	Education level^c^	>29%	Ref.	
		(Low volume)^•^	<14%	0.79 (0.70–0.89)	<0.001
			14–19.9%	0.92 (0.82–1.03)	
			20–28.9%	0.97 (0.86–1.08)	
Erhunmwunse (2017)	Esophageal	Education quartile	Q1^a^	Ref.	
			Q2	0.949 (0.872–1.033)	1
			Q3	0.914 (0.835–1.000)	0.39
			Q4^b^	0.834 (0.751–0.926)	0.009

CI, Confidence Interval; HR, Hazard Ratio; Q, Quartile; Ref., Reference. •Hospital by case volume-low: 1–99 cases per decade, middle: 100–200 cases per decade, and high: >200 cases per decade.

¥ Studies analyzing specifically gastric adenocarcinoma.

a Lowest.

b Highest.

c Education as reported by the NCDB is the percentage of adults in the area of residence of a given patient (based on zip code derived from the 2000 US census) who did not graduate from high school.

**Table 6:** Results of included studies assessing social and community context by cancer type (*n* = 4).

Author (year)	Cancer type	Category	Group	HR (95%-CI)	*p*-value
Xu (2022)	Gastric^¥^	Marital status	Married	Ref.	
			Divorced	1.111 (1.059–1.165)	<0.001
			Single	1.113 (1.071–1.156)	<0.001
			Widowed	1.180 (1.135–1.228)	<0.001
Qiu (2016)	Gastric^¥^	Marital status	Married	Ref.	
			Unmarried	1.09 (1.01–1.17)	0.027
Jin (2016)	Gastric	Marital status	Married	Ref.	
			Single (never married)	1.279 (1.216–1.344)	<0.001
			Divorced/separated	1.217 (1.149–1.290)	<0.001
			Widowed	1.274 (1.209–1.342)	<0.001
Wang (2019)	Duodenal	Marital status	Married	Ref.	
			Unmarried	1.259 (1.118–1.419)	<0.001

CI, Confidence Interval; HR, Hazard Ratio; Q, Quartile; Ref., Reference.

¥ Studies analyzing specifically gastric adenocarcinoma.

## 3 Results

Table 1 summarizes the descriptive characteristics of the evidence included in our review. We identified studies in GC (*n* = 12), with eight studies focused on gastric adenocarcinoma (GAC) and four studies including other histologic subtypes (e.g., squamous cell carcinoma, mucinous carcinoma, signet ring cell carcinoma). Two studies focused on esophageal cancer (*n* = 2) and one on duodenal cancer (*n* = 1). One study originated from a single medical institution (Boston Medical Center) and one study was from a health system (multi-hospital healthcare system affiliated with the University of Tennessee Health Science Center). The remaining studies utilized national databases, including The Surveillance, Epidemiology, and End Results (SEER; *n* = 9) and The National Cancer Data Base (NCDB; *n* = 3).

Nine categories of SDOH were assessed across the 14 studies, using different measurement tools for the five main domains. Healthcare access and quality (*n* = 11), and economic stability (*n* = 7) were the most commonly analyzed domains. Within them, insurance status (*n* = 8) and income level (*n* = 5) were the most frequently assessed categories (Table 2).

### 3.1 Healthcare access and quality

Eleven studies analyzed Health Care Access and Quality (17–27). The main categories assessed were insurance status (*n* = 8), access to treatment (*n* = 3), type of facility (*n* = 2), and hospital volume (*n* = 1; Table 3).

The correlation of insurance status with OS outcomes was explored in seven studies focused on GC and two studies on esophageal cancer. Uninsured patients had a worse OS probability. This trend was consistently observed across most of the studies evaluating insurance status (18, 21, 22, 24–27). Fang et al. reported inferior OS for uninsured versus privately insured patients (*p* = 0.02), although this difference was not statistically significant when considering only CSS (23). Similar results were found for esophageal cancer patients that underwent esophagectomy when comparing privately insured with not privately insured patients (*p =* 0.002) (24). Medicaid coverage was an independent prognostic indicator of inferior OS among patients with GC (*p*-value = 0.001) (18) and locally advanced GC who underwent gastrectomy (*p* = 0.02), compared to those with private insurance (23). A survival disadvantage (statistical trend) was observed for patients with GC on Non-Federal Health Insurance (*vs* those on Medicare-Medicaid; *p* = 0.051) (21). Private insurance was associated with superior OS for patients with GC specifically in low-volume centers (1–99 surgeries over the 10-year observation period; *p* = 0.013). For patients with esophageal cancer treated with esophagectomy, inferior OS was evident for uninsured patients in both low- (*p* = 0.009), middle- (100–200 surgeries; *p* = 0.002), and high-volume (>200 surgeries) centers (*p* = 0.007) (25).

Among studies assessing the type of facility (24, 25), one study in esophageal cancer found that receiving treatment at a community center (*p* = 0.007) was associated with poor OS (24). Treatment of GC patients undergoing gastrectomy at academic institutions had better OS at low-volume centers (*p* = 0.011) (25).

Treatment access was evaluated by three studies in GC (17, 19, 20). There was a higher probability of not receiving cancer-directed therapy for patients living in high poverty areas (*p* = 0.0001) (17). A second study found that lower education level was associated with lack of treatment, which in turn was linked with inferior OS (*p* < 0.001) (20). Poor compliance with surgical treatment was significantly more common among single and widowed GC patients. Patients with poor compliance in turn demonstrated worse survival outcomes compared with those in the surgical compliance group (*p* < 0.0001) and those in the non-surgical group (*p* < 0.0001) (19).

### 3.2 Assessing economic stability

Seven studies (17, 18, 21, 24–26, 28) analyzed economic stability by assessing income (*n* = 5) and poverty (*n* = 3) using different metrics (Table 4).

When income was used to measure Social Economic Stability (SES), a superior OS probability was identified for esophageal cancer patients in the highest quartiles of income (Q3 vs. Q1 *p* = 0.004; Q4 vs. Q1 *p* < 0.0001) (24).

For patients with GC, the highest income level (i.e., in the highest quartile) was associated with improved OS (*p* = 0.016) and CSS (*p* = 0.037) (18). There was an insignificant trend in the same direction for quartiles 2 and 3 compared to the lowest quartile. Higher median household income was also associated with higher OS rates (*p* = 0.0306) (28). Among lower and middle-volume centers, patients with the highest income had a better probability for OS (*p* < 0.001) (25). Using a different metric, a univariate analysis found a survival disadvantage for patients who resided in zip codes where >20% of the population lived in poverty (24.0 vs. 16.0 months; *p* = 0.006) (21).

No significant association was identified between living in high-poverty areas and survival outcomes in two studies analyzing GC patients undergoing surgical treatment (cancer-direct surgery, or surgery with curative intent) (17, 26). One found no correlation between income (above or below the median) and disease-specific survival (DSS; *p* = 0.859) in univariate analysis (26). In the second study, multivariate analysis revealed a non-statistical significant trend for the highest level of poverty associated with worse survival (*p* = 0.04) (17).

### 3.3 Assessing education access and quality

Four studies analyzed the education domain using education level quartiles (*n* = 2) or percent of population without a high school diploma (*n* = 2; Table 5) (18, 24, 25, 28). For patients with GC, both moderate and high education levels were significantly associated with superior OS (*p* = 0.001 and *p* = 0.005, respectively) and CSS probabilities (*p* < 0.001 and *p* = 0.004, respectively) (18). In another analysis, patients living in areas where a lower percent of the population did not have a high school education had OS probabilities (*p* = 0.0306) (28).

Among the studies that evaluated esophageal cancer OS outcomes, a higher level of education also had a positive impact (*p* = 0.009) (24), with better OS in low-volume hospitals with a lower percentage of patients who did not graduate from high school (*p* < 0.001) (25).

### 3.4 Assessing social and community context

Four studies analyzed marital status within the social and community context domain (Table 6) (18, 27, 29, 30). Being unmarried (including widowed/widower, single (never married) and separated/divorced) had a negative impact on OS (*p* < 0.001) (18, 29) and CSS (*p* = 0.027) among patients with GC (27). One study in patients with duodenal cancer also reported that unmarried status had an unfavorable correlation with OS and CSS (*p* < 0.001) (30).

## 4 Discussion

According to projected 2040 cancer burden statistics, the expected increase in cancer incidence correlates significantly with the Human Development Index (HDI), particularly in low and medium HDI countries (1). HDI dimensions overlap with SDOH, and our systematic review emphasizes the strong correlation of various SDOH domains in GC outcomes.

Past studies have shown a correlation between insurance status and stage at diagnosis of cancer (31). In our analysis, insurance status had a strong correlation with OS outcomes, with uninsured patients having a higher death risk compared to their insured counterparts. Interestingly, Medicaid-covered patients often experienced worse outcomes, possibly due to correlation with factors such as poverty, nutritional status, and lack of support systems among Medicaid beneficiaries (18, 23). Unfortunately, the unidimensional (or oligodimensional) evaluation of this metric does not allow proper evaluation of the whole universe of SDOH for these patients or the stage at diagnosis. The type of healthcare facilities where patients are treated also play a role, with academic institutions affiliated health facilities having the best OS (21, 25). The reasons for this are not explored in the studies analyzed, and may include more specialized and multidisciplinary care, access to clinical trials, and others. Socioeconomic factors such as poverty and lower education levels were linked to reduced access and adherence to treatment, resulting in inferior survival outcomes (17, 19, 20). Therefore, interventions that address disparities in health insurance coverage and access to specialized centers are key to improve outcomes across the whole spectrum of patients.

Economic stability influences GC survival, which can be attributed to its impact on healthcare access. Higher income level is associated with improved access to healthcare, preventive services, timely diagnosis, and treatment adherence (32). In our review, higher income levels were consistently associated with improved OS and CSS in both GC and esophageal cancer (18, 24). Understanding the correlation of economic stability with other SDOH may help target interventions for vulnerable populations where the impact may be greatest. Earlier studies reported associations between education and income, serving as indicators of SES and contributing to increasing the incidence of GC (33).

The association between education and GC survival may reflect the broader context of health behaviors and health literacy. Higher education levels are associated with better health-seeking behaviors (34). Advanced education consistently predicted better GC survival, showing superior OS and CSS. Notably, education remains significant even when considered alongside income in the same SES model (24). These findings align with the existing literature, indicating a strong correlation between education and health (18, 24, 25, 28). Improving health literacy in the general population and among patients with cancer should be prioritized by health authorities, health systems and healthcare providers to improve understanding of the disease and its treatment, eliminate stigmas, and provide resources to access the best possible care including clinical trials.

Several studies have suggested that social relationships have a considerable impact on mortality risk (35, 36). Marital status is widely used as a measure of social integration; however, an increasing number of studies document its contrasting effects, depending on the level of marital quality (36–38). Essential indicators of social and community well-being, such as social inclusion, quality of relationships and social support, have not been evaluated in the context of GC. The studies reviewed here were constrained to assessing only marital status as the lone social and community SDOH among patients with GI cancer. Single individuals exhibited poorer OS and CSS compared to their married counterparts. These results highlight the supportive role of social relationships in navigating and coping with cancer (18, 27, 29, 30). Further research should explore the dynamics of social relationships and their impact on cancer care, including other relationships that may be of value to an individual (e.g., friends, other family members), the role of social support, and the impact of community interventions on patients’ well-being, which may help develop patient-centered care approaches.

A major strength of this study lies in its ability to address gaps resulting from the limited literature specifically examining the association between individual or a few SDOH and a specific cancer, such as GC. In contrast, most of the existing literature tends to study cancer as a whole (39, 40). This provides strength for larger cohorts but potentially overlooks or dismisses specific characteristics unique to certain cancers, such as GC.

Despite the strengths of our analysis, there are important limitations that should be noted. First, most studies used large national databases (SEER and NCDB). These databases are limited in data at an individual level, thus making generalizations that may not be applicable to individual patients. Further, these registries usually do not collect detailed information on SDOH. Second, the use of different definitions and classifications of SDOH and the use of different measurement tools results in difficulties in homogenizing the data. There are, for example, different approaches to analyze SES (above and below median vs. quartiles) or education (no high-school vs. other approaches). This should serve as a call for developing a more uniform and systematic approach to collecting data on SDOH both at individual institutions and in national databases.

## 5 Conclusion

Understanding SDOH in association with GI cancer survival requires exploring their underlying causes. Despite gaps in our knowledge of issues relating to the SDOH, as well as their reported heterogeneity, the impact of SDOH on cancer outcomes is clear. In order to address healthcare disparities in GC (and cancer in general), a comprehensive approach in collecting, reporting and analyzing SDOH is required. These factors should be included in outcomes analyses together with biologic characteristics and therapeutic interventions. A comprehensive approach would be better suited to understanding the correlation of all factors affecting outcomes in individual patients, and developing strategies to eliminate disparities. In the era of personalized medicine, we should not ignore that an important component of this personalization is the environment in which the patient lives.

## Data Availability

The original contributions presented in the study are included in the article/Supplementary material, further inquiries can be directed to the corresponding authors.

## Footnotes

^1^ https://www.crd.york.ac.uk/prospero/
