# Supplementary material for: Social determinants of health and upper gastrointestinal cancer outcomes in the United States: a systematic review
Source: Front Public Health. 2024 Nov 19;12:1477028. doi: 10.3389/fpubh.2024.1477028 (PMC11613319; doi:10.3389/fpubh.2024.1477028)
Supplement: Supplementary file 1 [file Table_1.DOCX]

Supplementary Material

| **Supplementary Table 1.** Search strategies related to the accessed database. | |
| --- | --- |
| **Database** | **Search** |
| **PubMed** | ("Cancer"[tiab]) AND ("Disparit*"[tiab] OR '"Social determin*"[tiab] OR "Socio*economic*"[tiab] OR "Social Determinants of Health"[Mesh]) AND ("Survival Analysis"[Mesh] OR "Surviv*"[Title/Abstract] OR "Recurre*"[Title/Abstract] OR "Relapse"[Title/Abstract] OR "Prognos*"[Title/Abstract] OR “Mortalit*” OR “Outcome*”[Title/Abstract]). |
| **Cochrane** | ("Cancer"[ti,ab,kw]) AND ("Disparit*"[ti,ab,kw] OR '"Social determin*"[ ti,ab,kw] OR "Socio*economic*"[ ti,ab,kw] OR "Social Determinants of Health"[Mesh]) AND "Surviv*" OR  "Recurre*" OR "Relapse" OR "Prognos*" OR “Mortalit*” OR “Outcome*”). |
| **EMBASE** | ('cancer':ab,ti) AND ('disparit*':ab,ti OR 'social determin*':ab,ti OR 'socio*economic*':ab,ti OR 'social determinants of health':ab,ti) AND (‘Survival Analysis’:ab,ti OR ‘Surviv*’:ab,ti OR ‘Recurre*’:ab,ti OR ‘Relapse’:ab,ti OR ‘Prognos*’:ab,ti OR ‘Mortalit*’ OR ‘Outcome*’:ab,ti) AND  [embase]/lim AND ([article]/lim OR [article in press]/lim). |
| **SCOPUS** | TITLE-ABS("cancer") AND TITLE-ABS("disparit*" OR "Social determinan*" OR "Socio*economic*"OR "Social Determinants of Health") AND TITLE-ABS("Survival Analysis" OR "Surviv*" OR "Recurre*" OR "Prognos*" OR "Mortalit*" OR "outcome*") AND ( LIMIT-TO ( DOCTYPE,"ar" ) )  AND ( LIMIT-TO ( LANGUAGE,"English" ) ) |
| **Web of Science** | (TI=(Cancer) OR AB=(Cancer)) AND (TI=("disparit*" OR "Social determinan*" OR "Socio*economic*" OR "Social Determinants of Health") OR AB=("disparit*" OR "Social determinan*" OR "Socio*economic*" OR "Social Determinants of Health")) AND (TI=("Survival Analysis" OR "Surviv*" OR "Recurre*" OR "Prognos*" OR "Mortalit*" OR "outcome*") OR AB=("Survival Analysis" OR "Surviv*" OR "Recurre*" OR "Prognos*" OR "Mortalit*" OR  "outcome*")) |
| **Google Scholar** | In the title of the article: Cancer AND "Social Determinants" |

| **Supplementary Table 2.** Studies excluded and reason categorization ***(n*=10).** | | |
| --- | --- | --- |
| **Exclusion**  **category** | **Studies excluded (Author, year)** | **Studies**  **N** |
| **Reason 1** | Antunez, A. G. et al. 2019 (1), Schrag,D. et al.2002 (2), Patel, K. S. et al.2020 (3), Robbins, A. S. et al.2010 (4), Lin, M. et al.2022 (5), Nitzkorski, J. R. etal. 2013 (6). | 6 |
| **Reason 2** | Ho, M. Y. et al,2014 (7), Lamm, R.et al,2022 (8) , Sun, F. et al 2018 (9). | 3 |
| **Reason 3** | Melillo, A. et al. 2020 (10). | 1 |
| Reason 1, Colorectal Cancer; Reason 2, Outcome measures other than survival; Reason 3, Bladder Cancer. | |  |

**References**

1. Antunez A. G., Kanters A. E., Regenbogen S. E. Evaluation of Access to Hospitals Most Ready to Achieve National Accreditation for Rectal Cancer Treatment. JAMA Surg. 2019;154:516-23.

2. Schrag Deborah*†. Hospital and Surgeon Procedure Volume as Predictors of Outcome Following Rectal Cancer Resection. Annals of Surgery. 2002;236:583–92.

3. Patel K. S., Alhatem A., Gadde U., al. e. Insurance status and level of education predict disparities in receipt of treatment and survival for anal squamous cell carcinoma. Cancer Epidemiol. 2020;67:101723.

4. Robbins AS, Chen AY, Stewart AK, al. e. Insurance status and survival disparities among nonelderly rectal cancer patients in the National Cancer Data Base. Cancer. 2010;116:4178-86.

5. Lin M., O'Guinn M., Zipprer E., al. e. Impact of Medicaid Expansion on the Diagnosis, Treatment, and Outcomes of Stage II and III Rectal Cancer Patients. J Am Coll Surg. 2022;234:54-63.

6. Nitzkorski J. R., Willis A. I., Nick D. Association of race and socioeconomic status and outcomes of patients with rectal cancer. Ann Surg Oncol. 2013;20:1142-7.

7. Ho M. Y., Al-Barrak J., Peixoto R. D., al. e. The association between county-level surgeon density and esophageal and gastric cancer mortality. J Gastrointest Cancer. 2014;45:487-93.

8. Lamm R., Hewitt D. B., Li M. Socioeconomic Status and Gastric Cancer Surgical Outcomes: A National Cancer Database Study. J Surg Res. 2022;275:318-26.

9. Sun F., Sun H., Mo X. Increased survival rates in gastric cancer, with a narrowing gender gap and widening socioeconomic status gap: A period analysis from 1984 to 2013. J Gastroenterol Hepatol. 2018;33:837-46.

10. Melillo A., Linden K., Spitz F. Disparities in Treatment for Gallbladder Carcinoma: Does Treatment Site Matter? J Gastrointest Surg. 2020;24:1071-6.
